# Pancreatic Divisum: An Unusual Cause of Chronic Pancreatitis in a Young Patient

**DOI:** 10.7759/cureus.1856

**Published:** 2017-11-17

**Authors:** Aaron R Kuzel, Muhammad Uzair Lodhi, Mustafa Rahim

**Affiliations:** 1 Department of Emergency Medicine, Lincoln Memorial University-Debusk College of Osteopathic Medicine; 2 Medical Student, Department of Medicine, Raleigh General Hospital, Beckley, Wv; 3 Assistant Clinical Professor of Internal Medicine, West Virginia University School of Medicine

**Keywords:** acute pancreatitis, chronic pancreatitis, type iii pancreatic divisum, pancreatic divisum, abdominal pain, mrcp, recurrent pancreatitis

## Abstract

Pancreatic divisum is a condition that occurs in 4-14% of the population. Pancreatic divisum occurs in development when the ventral bud and dorsal bud of the pancreas fail to fuse. Patients with this condition are usually asymptomatic, however, 25-38% of these patients experience recurrent pancreatitis that may further progress to chronic pancreatitis. This case is of a 20-year-old female presenting with abdominal pain in the left and right upper quadrants of the abdomen with a significant history of recurrent pancreatitis since the age of seven. The patient was examined with computed tomography (CT), which identified pancreatitis. Further magnetic resonance cholangiopancreatography (MRCP) assisted in the diagnosis of a type III pancreatic divisum, given the remnant of short communication between the dorsal and ventral duct.

## Introduction

Pancreatic divisum is a congenital anomaly occurring in 4-14% of the population [1]. In normal embryological development, the ventral bud of the pancreas rotates around the foregut allowing the dorsal and ventral pancreas to fuse. The ventral pancreatic duct further fuses with the dorsal pancreatic duct to form the duct of Wirsung or main pancreatic duct, which later joins the common bile duct and drains into major papilla. The majority of normal pancreas drains through this main pancreatic duct. The dorsal duct in turn remains as the duct of Santorini, which empties into the duodenum through the minor papilla [2]. Pancreatic divisum, however, occurs when the ventral and dorsal buds fail to fuse. When this occurs, the majority of the pancreas drains through the dorsal duct, also referred to as the duct of Santorini, into the minor papilla [1]. There are three major types of pancreatic divisum. Type I, or classic pancreatic divisum, is a complete failure of the dorsal and ventral buds to fuse. Type II pancreatic divisum is characterized by the absence of the ventral duct, so the minor papilla drains the entire pancreas and the major papilla drains some of the common bile duct. Finally, type III presents with a small remnant communication between the dorsal duct and ventral duct [3]. The majority of cases of pancreatic divisum are asymptomatic, but there has been a reported frequency of acute pancreatitis as a result of pancreatic divisum ranging between 25-38% [1-2]. Often these patients experience recurrent pancreatitis which could develop into a chronic pancreatitis.

## Case presentation

A 20-year-old female with significant past medical history of recurrent pancreatitis presented to the emergency department with abdominal pain that was radiating to the back, to the left inferior angle of the scapula. She described it as sharp, stabbing pain of 8/10 on severity scale. She mentioned that she has experienced this type of pain before, but it has been three years since her last episode. She was unable to consume solid foods without “debilitating” nausea, but denied vomiting. The patient mentioned that her first episode of pancreatitis was at the age of seven when she underwent surgery for pancreatic cyst drainage. Family history was also unremarkable. She has an 11 pack year smoking history but denied alcohol intake. Her vitals were as follow: blood pressure of 132/80 mmHg, heart rate of 81 bpm, 94% oxygen saturation, and a temperature of 98.6 F. Patient weighed 247 lbs with a BMI of 36.49. Abdominal examination revealed a large scar in the center of the abdomen with bowel sounds present in all quadrants. Her abdomen was soft, and non-distended but presented with guarding and tenderness, which was especially tender in the left and right upper quadrants radiating to the umbilicus. She had a negative Murphy sign. Her labs were consistent with elevated lipase and amylase. Computed tomography examination conducted in 2010 and 2017 showed evidence of pancreatitis. In the CT study performed in 2017, there was a 10-mm calcification discovered in the head of the pancreas that was not seen in the 2010 study. We suggested magnetic resonance cholangiopancreatography (MRCP) radiological analysis for the patient which displayed a dilated pancreatic head with a maximal diameter of 6-mm. The MRCP findings demonstrated a small amount of fluid between the pancreatic head and proximal descending duodenum, as well as the posterior border of the liver and diaphragm consistent with pancreatitis. The MRCP also showed evidence of a small remnant communication between the dorsal duct (which becomes the main source of drainage in pancreatic divisum) and the ventral duct.

## Discussion

**Figure 1:**
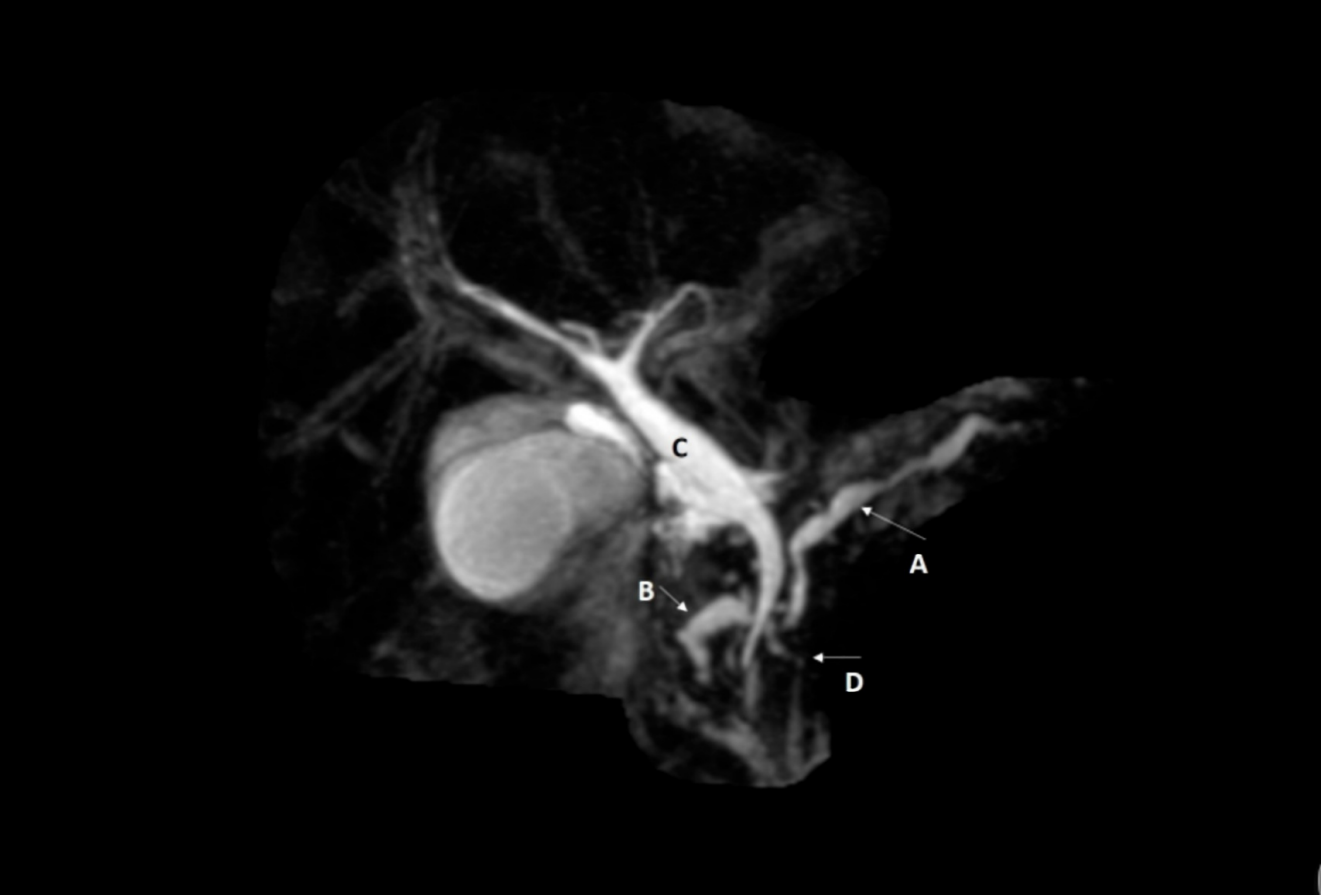
Magnetic resonance cholangiopancreatography (MRCP) of the abdomen showing pancreas divisum. (A) Indicates the dorsal pancreatic duct (which is the most prominent duct in pancreatic divisum) as it crosses over the common bile duct. There are obvious dilatations of this dorsal pancreatic duct as shown in this MRCP, the largest being 6.1 mm, consistent with chronic pancreatitis. (B) Indicates part of the dorsal duct, where it drains directly into the duodenum through minor papilla. The inherently small diameter of the minor papilla causes increased pressure in the dorsal pancreatic duct, as shown in this MRCP. (C) Indicates the common bile duct, which is joined by ventral pancreatic duct before draining into duodenum through the major papilla. (D) Indicates the short remnant or filamentous communication between the ventral and the dorsal duct that define this as a type III pancreatic divisum.

Acute pancreatitis is defined as the presence of any two symptoms including: abdominal pain radiating to the back, serum lipase >180 U/L, and radiological evidence of inflammation of the pancreas. Recurrent pancreatitis is defined as two or more episodes of acute pancreatitis. Chronic pancreatitis can be diagnosed with the presence of calcifications on computed tomography [4]. Given the patient has had several episodes of pancreatitis, two of those episodes in our case; the patient’s diagnosis is consistent with recurrent pancreatitis. In addition, the CT study performed in 2017 exhibited a 10-mm calcification in the head of the pancreas that is consistent with chronic pancreatitis. To rule out cystic fibrosis as the recurrent cause of pancreatitis, a sweat chloride test and CFTR genetic testing were administered but returned negative [5]. Given the patient’s family medical history is negative for cystic fibrosis as well as any medical issues involving the pancreas, the patient is unlikely to have cystic fibrosis or hereditary pancreatitis. While the patient’s recurrent pancreatitis and progression to chronic pancreatitis is consistent with the congenital nature of hereditary pancreatitis, this condition has autosomal dominant transmission. Since the patient’s mother, father, or sibling denied any pancreatic problems, this diagnosis of hereditary pancreatitis is less likely [6]. The patient also presented with right upper quadrant tenderness and given her high BMI, the patient was examined for choledocholithiasis. These tests returned negative as an abdominal ultrasound displayed no signs of gallstones or any acute inflammation of the gallbladder. Further MRCP analysis exhibited no evidence of stones. Magnetic resonance cholangiopancreatography or MRCP is a non-invasive radiological study that can visualize the pancreatic ducts without the use of contrast material. MRCP scan is the most sensitive test for pancreatic divisum [2]. In this case, MRCP showed evidence of a type III pancreatic divisum as evidenced by a short communication existing between the dorsal (the most prominent duct) and ventral duct. In addition, MRCP also exhibited dilatations of the dosal duct showing chronic pancreatitis (Figure 1). The dorsal duct as it failed to fuse with the ventral duct, drains directly into the duodenum through the minor papilla. While the ventral duct joins the common bile duct and drains into the duodenum through the major papilla. The dorsal duct functions as a major source of drainage in pancreatic divisum, as shown below (Figure 1). The relatively small diamater of the minor papilla increases the pressure in dorsal pancreatic duct, resulting in obstruction of pancreatic exocrine secretions, ductal distention and recurrent pancreatitis. 

The patient was instructed for smoking cessation, to avoid alcohol use, and was told to remain on a strict low-fat diet to avoid any further exacerbation of pancreatitis. The patient will be monitored every six months for any signs of acute pancreatitis recurrence, exacerbation of her chronic pancreatitis, or progression to pancreatic cancer. For patients with abdominal pain and recurrent pancreatitis as a result of pancreatic divisum, there is also a corrective surgical option. The patient may elect to receive endoscopic stenting or sphincterotomy of a minor papilla [2].

Lastly, detailed review of the patient’s past medical history revealed that each of the past recurrent episodes of pancreatitis were treated conservatively and discharged upon resolution of symptoms. Performance of detailed imaging studies during the patient's initial hospital course may have prevented further complications. Had the patient been assessed for recurrent episodes of pancreatitis with detailed radiological imaging upon initial hospital admissions, it is likely that the congenital defect could have been diagnosed earlier, therefore preventing the chronic deterioration of the pancreas and additional hospital admissions.

## Conclusions

In this case report, we attempted to highlight that a young patient presenting with recurrent pancreatitis is suspicious for many pathological processes. Of course, when approaching a patient with these symptoms and confirmed radiological evidence of chronic pancreatitis, it is recommended to rule out cystic fibrosis or alcohol-related causes. However, should these pathologies be negative or not comprehensive in explanation of the patient’s problem, then clinicians should strongly consider the possibility of pancreatic divisum, especially if the family history is negative for recurrent pancreatitis.
